# Immune combinational therapy targeting OX40 and IDO synergistically enhances efficacy of a cancer vaccine

**DOI:** 10.1186/2051-1426-2-S3-P226

**Published:** 2014-11-06

**Authors:** Zuzana Berrong, Shamim Ahmad, Rasha Abu Eid, Abdeljabar El Andaloussi, Tanusree Sen, Ross Stewart, Scott A Hammond, Rajeev Shrimali, Mikayel Mkrtichyan, Samir N Khleif

**Affiliations:** 1 Cancer Center, Georgia Regents University Cancer Center, Augusta, GA, USA; 2 MedImmune, Cambridge, UK; 3 Medimmune LLC, Gaithersburg, MD, USA

## 

Enhancing an effector immune response by cancer vaccines has not been clinically successful so far. Although the necessary immune response may be elicited using vaccine-based therapies, this has not been sufficient for a positive clinical outcome; since most cancers can escape immune surveillance via multiple immune suppressive mechanisms induced in the tumor environment. A relatively recent strategy to increase the therapeutic efficacy of tumor vaccination is to combine it with alternative immunotherapeutic approaches.

Here we tested if simultaneous targeting of the effector arm of the immune system with an agonist anti-OX40 mAb together with targeting of suppressive mechanisms in the tumor, via inhibition of indoleamine-(2,3)-dioxygenase (IDO), could enhance the anti-tumor activity of vaccine in mouse models of cancer.

The OX40 molecule is a co-stimulatory receptor expressed on T-cells that can lead to proliferation and enhancement of T-cell effector function when triggered with an agonistic antibody. However, the tumor secretes a number of suppressive signals as a protective mechanism against its destruction, one of which is IDO. We demonstrate that treatment of TC-1 tumor bearing mice with an anti-OX40 agonist antibody in combination with vaccine leads to the enhancement of antigen-specific T cell responses. The dose of 1 mg/kg anti-OX40 antibody stimulated the effector arm of T cells, which ultimately led to a significant increase in the CD8^+^/regulatory T-cell (Treg) ratio within tumors. Further, this combination of vaccine and anti-OX40 antibody treatment led to a significant inhibition of tumor growth and to a prolonged survival of tumor bearing mice compared to control, and resulted in complete tumor regression in 20% of treated mice. We found, however, that this anti-tumor activity could be further enhanced through the combination of vaccine, anti-OX40 and 1-methyltryptophan (1-MT), an IDO activity inhibitor. IDO has been shown to be secreted by tumor cells, suppressive dendritic cells and macrophages in tumor environment, and is known to be responsible for suppressing effector cells and inducing Tregs.

Our data demonstrate that vaccine/anti-OX40 antibody/1-MT combination leads to a more profound inhibition of tumor growth in mice and complete regression of established tumors in 60% of treated mice. In conclusion, we provide evidence that simultaneous targeting of the effector arm of the immune system, via anti-OX40 antibody, and suppressive mechanisms within the tumor, via 1-MT, in combination with cancer vaccine has a synergistic effect for tumor eradication and is a promising strategy that could potentially enhance the overall efficacy of cancer treatment in patients.

